# A Roadmap to Gene Discoveries and Novel Therapies in Monogenic Low and High Bone Mass Disorders

**DOI:** 10.3389/fendo.2021.709711

**Published:** 2021-08-13

**Authors:** Melissa M. Formosa, Dylan J. M. Bergen, Celia L. Gregson, Antonio Maurizi, Anders Kämpe, Natalia Garcia-Giralt, Wei Zhou, Daniel Grinberg, Diana Ovejero Crespo, M. Carola Zillikens, Graham R. Williams, J. H. Duncan Bassett, Maria Luisa Brandi, Luca Sangiorgi, Susanna Balcells, Wolfgang Högler, Wim Van Hul, Outi Mäkitie

**Affiliations:** ^1^Department of Applied Biomedical Science, Faculty of Health Sciences, University of Malta, Msida, Malta; ^2^Centre for Molecular Medicine and Biobanking, University of Malta, Msida, Malta; ^3^School of Physiology, Pharmacology, and Neuroscience, Faculty of Life Sciences, University of Bristol, Bristol, United Kingdom; ^4^The Musculoskeletal Research Unit, Translational Health Sciences, Bristol Medical School, Faculty of Health Sciences, University of Bristol, Bristol, United Kingdom; ^5^Department of Applied Clinical Sciences and Biotechnological, University of L’Aquila, L’Aquila, Italy; ^6^Department of Molecular Medicine and Surgery, Karolinska Institutet, Stockholm, Sweden; ^7^Department of Clinical Genetics, Karolinska University Hospital, Stockholm, Sweden; ^8^IMIM (Hospital del Mar Research Institute), Centro de Investigación Biomédica en Red de Fragilidad y Envejecimiento Saludable (CIBERFES), Barcelona, Spain; ^9^Department of Internal Medicine, Erasmus University Medical Center, Rotterdam, Netherlands; ^10^Department of Genetics, Microbiology and Statistics, Faculty of Biology, Universitat de Barcelona, Centro de Investigación Biomédica en Red de Enfermedades Raras (CIBERER), Institut de Biomedicina de la Universitat de Barcelona (IBUB), Institut de Recerca Sant Joan de Déu (IRSJD), Barcelona, Spain; ^11^Molecular Endocrinology Laboratory, Department of Metabolism, Digestion and Reproduction, Imperial College London, London, United Kingdom; ^12^Department of Surgery and Translational Medicine (M.L.B.), University of Florence, Florence, Italy; ^13^Department of Medical Genetics and Skeletal Rare Diseases, IRCCS Rizzoli Orthopaedic Institute, Bologna, Italy; ^14^Department of Paediatrics and Adolescent Medicine, Johannes Kepler University Linz, Linz, Austria; ^15^Institute of Metabolism and Systems Research, University of Birmingham, Birmingham, United Kingdom; ^16^Department of Medical Genetics, University of Antwerp, Antwerp, Belgium; ^17^Children’s Hospital, University of Helsinki and Helsinki University Hospital, Helsinki, Finland; ^18^Research Program for Clinical and Molecular Metabolism, Faculty of Medicine, University of Helsinki, Helsinki, Finland; ^19^Folkhälsan Research Centre, Folkhälsan Institute of Genetics, Helsinki, Finland

**Keywords:** bone mass, monogenic bone disorders, gene variants, functional validation, drug discovery, GEMSTONE, skeletal dysplasia

## Abstract

Genetic disorders of the skeleton encompass a diverse group of bone diseases differing in clinical characteristics, severity, incidence and molecular etiology. Of particular interest are the monogenic rare bone mass disorders, with the underlying genetic defect contributing to either low or high bone mass phenotype. Extensive, deep phenotyping coupled with high-throughput, cost-effective genotyping is crucial in the characterization and diagnosis of affected individuals. Massive parallel sequencing efforts have been instrumental in the discovery of novel causal genes that merit functional validation using *in vitro* and *ex vivo* cell-based techniques, and *in vivo* models, mainly mice and zebrafish. These translational models also serve as an excellent platform for therapeutic discovery, bridging the gap between basic science research and the clinic. Altogether, genetic studies of monogenic rare bone mass disorders have broadened our knowledge on molecular signaling pathways coordinating bone development and metabolism, disease inheritance patterns, development of new and improved bone biomarkers, and identification of novel drug targets. In this comprehensive review we describe approaches to further enhance the innovative processes taking discoveries from clinic to bench, and then back to clinic in rare bone mass disorders. We highlight the importance of cross laboratory collaboration to perform functional validation in multiple model systems after identification of a novel disease gene. We describe the monogenic forms of rare low and high rare bone mass disorders known to date, provide a roadmap to unravel the genetic determinants of monogenic rare bone mass disorders using proper phenotyping and genotyping methods, and describe different genetic validation approaches paving the way for future treatments.

## Introduction

Skeletal development is regulated by numerous genetic factors that guide the growth, modeling and remodeling of skeletal structures starting in early fetal development and continuing throughout life. These processes are crucial for attainment of normal height, skeletal patterning, bone shape, and mobility, but also for maintenance of normal bone mass and fracture resistance. Defects in the involved genes result in a large and heterogeneous group of disorders, collectively called skeletal dysplasias, in which the primary features are confined to the skeleton. More than 460 different forms of skeletal dysplasia, most of them monogenic, have been recognized (1). They are estimated to affect approximately 1/5,000 children (2, 3), and can have distinct clinical manifestations and course. Clinical outcomes range in severity from neonatal lethality to only mild growth retardation, deformity or fracture risk. Diagnosis is based on growth pattern and other clinical characteristics, skeletal imaging, bone density testing, biochemical diagnostics, and genetic tests. Although the genetic basis has been described and mutations in the responsible genes identified in a significant proportion of these conditions, for several distinct skeletal dysplasia phenotypes the genetic cause is still not known (1).

Within this large group of genetic skeletal disorders, monogenic disorders affecting bone mass comprise an expanding subgroup (1, 4). This includes disorders with low bone mass and skeletal fragility, and disorders leading to increased bone mass, both commonly associated with extra-skeletal complications (5, 6). Due to significant variability in severity, diagnosis can be challenging. Importantly, the underlying molecular genetic mechanisms for these disorders remain inadequately explored and, in several entities, the causative genetic defect, and underlying cellular and molecular pathophysiology are still uncharacterized.

The various skeletal dysplasia delineated to date have provided important information about the molecular pathways governing skeletal health both in these conditions and in the general population, underscoring the significance of new gene discoveries not only for the individuals affected by the monogenic rare bone mass disorder, but also more widely to the musculoskeletal research field (7). Indeed, the large wealth of data generated from monogenic and polygenic bone mass disorders, frailty and other musculoskeletal traits, have led to establishment of GEMSTONE, a COST action (CA18139; https://cost-gemstone.eu) set up to bring together multidisciplinary researchers actively involved in the musculoskeletal field. The aim of GEMSTONE is to translate the genetic discoveries into meaningful clinical applications and personalized medicine, discussed in more detail in Koromani et al., “GEnomics of MusculoSkeletal traits TranslatiOnal NEtwork (GEMSTONE): origins, rationale, organization and prospects” (8).

This paper aims to provide an overview of the presently known monogenic disorders of low and high bone mass, and to provide a roadmap to move forward in identifying and characterizing novel genetic forms, with the aim of utilizing these discoveries to develop novel therapies. We have elucidated the various steps of this process: deep phenotyping of the patient with a potentially novel skeletal condition, genetic evaluation and discovery of the causative gene defect, functional *in vitro* and *in vivo* validation, and potential approaches for novel drug discoveries.

## The Spectrum of Monogenic Bone Fragility Disorders

### Origins of Bone Fragility

The composition, amount and microarchitecture of human bone tissue determines its fracture load and resistance to bending and torsional forces. Increased bone fragility with low bone mass is either acquired (most commonly secondary to medications e.g., corticosteroids, aging, immobility, low body weight, nutritional deficiencies, systemic disorders e.g., chronic inflammatory conditions, endocrine pathology), or genetic, or a multitude of both (7, 9). Relevant to GEMSTONE, more specifically **Working Group 3** (**Monogenic conditions – human knockout models**), are the rare monogenetic forms of low and high bone mass, that result from mutations in genes regulating bone composition and function of essential proteins required for type I collagen assembly, osteoblastic bone formation, bone mineralization and osteoclastic bone resorption. The advancements in sequencing methods, especially massively parallel sequencing, have enabled identification of many new monogenic disorders caused by defects in genes that encode proteins in various metabolic pathways (10).

### Monogenic Low Bone Mass Disorders

Osteogenesis imperfecta (OI) is, by far, the most common heritable cause of increased bone fragility. It is characterized by fractures, often at a young age, low bone mass, deformities and variable short stature, but also by a number of extra-skeletal features such as blue sclerae, dental problems, skin and joint laxity, and a hearing deficit (11). Currently, more than 20 distinct genetic entities have been grouped under this diagnosis (12). They have in common that they all, *via* different mechanisms, affect the quality or quantity of type I collagen, the main protein component of the skeleton. However, the spectrum of mechanisms ultimately leading to defective collagen assembly in the extracellular matrix has expanded considerably recently, necessitating several revisions in classifications (13, 14). In most cases, studies on disease mechanisms *in vitro* and in animal models have indicated defects in collagen secretion and structure, procollagen transport, folding, post-translational modification, processing and crosslinking. By contrast, some forms of OI are associated with impaired mineralization (*IFITM5*, *SERPINF1*), or defective osteoblast differentiation and function (*SP7, WNT1*), rather than a direct defect in type I collagen deposition (13, 15).

Clinically, the variability of presentation and genotypes has made it difficult to differentiate between OI and so-called primary osteoporosis, which is why we present a spectrum of these entities (**Table 1**). For example, non-collagen pathways associated with bone fragility include the osteoblastic WNT signaling pathway (e.g., *WNT1, LRP5*) which regulates bone formation and the OPG/RANKL system (e.g., *TNFRSF11A*) which regulates osteoclast activity. Various forms of OI and primary osteoporosis are still incompletely understood; the extremely rare conditions associated with bone fragility continue to hold secrets to our understanding of bone fragility which future research will need to explore (13, 15).

**Table 1:** Bone fragility conditions with low and high bone mass; the gene defects, function and clinical characteristics.

Condition	OMIM	Inheritance	Gene	Mutation	Protein	Function	Symptoms*
**Osteogenesis imperfecta & Primary Osteoporosis**	166200 166210 259420 166220	AD	*COL1A1 COL1A2*	LoF	Collagen alpha-1(I) chain Collagen alpha-2 (I) chain	Collagen synthesis	Mild form, Clinical type I Perinatal lethal, Clinical type II Severe form, Clinical type III Moderate form, Clinical type IV
	610967	AD	*IFITM5*	GoF	Interferon-induced Transmembrane protein 5 (BRIL)	Mineralization	Clinical types V; and III (atypical type 6)
	613982	AR	*SERPINF 1*	LoF	Pigment epithelium-derived factor (PEDF)	Mineralization	Clinical type III
	610854	AR	*CRTAP*	LoF	Cartilage-associated protein (CRTAP)	Collagen modification	Clinical types II, III, IV
	610915	AR	*LEPRE1 (P3H1)*	LoF	Leucine proline enrichedproteoglycan1 / Prolyl 3-hydroxylase 1 (P3H1)	Collagen modification	Clinical types II, III
	259440	AR	*PPIB*	LoF	Cyclophilin B (CyPB)	Collagen modification	Clinical types II, III
	613848	AR	*SERPINH1*	LoF	Serpin peptidase inhibitor, clade H, member 1/heat shock protein 47	Collagen folding and cross-linking	Clinical type III
	610968 259450	AR AR	*FKBP10*	LoF	Peptidyl-prolyl cis-transisomerase FKBP10	Collagen folding and cross-linking	Clinical types III, IV Bruck Syndrome Type 1
	613849	AR	*SP7*	LoF	Zinc-finger transcription factor, Osterix	Osteoblast differentiation and maturation	Clinical type IV
	112264	AR	*BMP1*	LoF	Bone morphogenic protein1/ procollagen C proteinase	Collagen processing	Clinical Type III
	615066	AR	*TMEM38B*	LoF	Trimeric intracellular cation channel B (TRIC-B)	ER calcium flux	Clinical type I, III, IV
	615220	AR AD	*WNT1*	LoF	Wingless-type MMTV integration site family, member 1	WNT signaling	Clinical type III, IV Primary osteoporosis
	616229	AR AD	*CREB3L1*	LoF	Old astrocyte specificallyinduced substance (OASIS)	ER UPR response, ER-Golgi trafficking, cleavage stimulated by ceramide	Clinical type III Clinical type I
	616507	AR	*SPARC*	LoF	Secreted protein, acidic, cysteine-rich (SPARC, or osteonectin)	Procollagen processing and extracellular assembly	Clinical type III, IV
	617952 601559	AR	*TENT5A (FAM46A)*	LoF	Terminal nucleotidyltransferase 46, Member A (FAM46A)	BMP signaling	Clinical type III, overlap with Stuve-Wiedemann syndrome
	301014	XR	*MBTPS2*	LoF	Site 2 protease (S2P)	Golgi Regulated intramembrane proteolysis	Clinical type III, IV
	607783	AR	*MESD*	LoF	Mesoderm development LRP chaperon	WNT signaling	Clinical type III
	607186	AR	*SEC24D*	LoF	SEC24D	ER COPII transport of procollagen	Clinical type III, overlap with Cole-Carpenter Syndrome 2
	618788	AR	*CCDC134*	LoF	Coiled-coil domain containing 134	MAPK pathway	Clinical type III
	609024	AR	*KDELR2*	LoF	KDEL endoplasmic reticulum protein retention receptor 2	Collagen fibre formation	Clinical type II, III
**Other Primary Osteoporosis**	259770 166710	AR AD	*LRP5*	LoF	Low density lipoprotein receptor 5 (LRP5)	WNT signaling	Osteoporosis pseudoglioma syndrome Primary osteoporosis
	300910	XL	*PLS3*	LoF	Plastin 3	Formation of F-actin bundles	Primary osteoporosis
	609220	AR	*PLOD2*	LoF	Telopeptide lysyl hydroxylase	Collagen crosslinking	Bruck Syndrome 2
	126550	AD	*SGMS2*	LoF	Phosphatidylcholine:ceramide Cholinephosphotransferase 2	Mineralization	Calvarial doughnut lesions with bone fragility without (CDL) or with spondylometaphyseal dysplasia (CDLSMD)
	112240	AD	*P4HB*	LoF	Protein disulfide-isomerase	Catalyzes rearrangement of disulfide bonds	Cole-Carpenter syndrome 1
	605822	AR	*XYLT2*	LoF	Xylosyltransferase 2	Proteoglycan biosynthesis	Spondylo-ocular dysplasia
	166260	AD	*ANO5*	LoF	Anoctamin-5	Unclear (chloride channel)	Gnathodiaphyseal dysplasia
	231070	AR	*GORAB*	LoF	RAB6-interacting golgin	Unclear	Geroderma osteodysplasticum
	612940	AR	*PYCR1*	LoF	Pyrroline-5-carboxylate reductase 1, mitochondrial	Unclear (Prolin biosynthesis)	Cutis laxa (ARCL2B)
	182250	AD	*IFIH1*	GoF	Interferon-induced helicase C domain-containing protein 1	Unclear (Antiviral innate immunity)	Singleton-Mertin dysplasia Type 1
	616298	AD	*DDX58*	GoF	Antiviral innate immune response receptor RIG-I	Unclear (antiviral innate immunity)	Singleton-Mertin dysplasia Type 2
	616866	AR	*TRIP4*	LoF	Activating signal cointegrator 1	Unclear (transcription coactivator)	Spinal muscular atrophy with congenital bone fractures-1 (SMABF1)
	616867	AR	*ASCC1*	LoF	Activating signal cointegrator 1 complex subunit 1	Unclear (DNA damage repair)	Spinal muscular atrophy with congenital bone fractures-2 (SMABF2)
**Osteolysis group**	174810 602080	AD	*TNFRSF11A*	GoF	Tumor necrosis factor receptor superfamily member 11A	Increased RANKL-mediated osteoclastogenesis	Familial expansile osteolysis (FEO) Juvenile Paget’s Disease (PDB2)
	259600 277950	AR	*MMP2* *MMP14*	LoF	Matrix metalloproteinase 2Matrix metalloproteinase 14	Unclear (collagenolysis)	Multicentric osteolysis, nodulosis and arthropathy (MANO)
	102500	AD	*NOTCH2*	GoF	Neurogenic locus notch homolog protein 2	Regulate cell fate; osteoblast and osteoclast function	Hajdu-Cheney Syndrome
**Demineralization group**	241500 146300	AR AD	*TNSALP*	LoF	Tissue non-specific alkaline phosphatase	Mineralization	Hypophosphatasia
	307800	XL	*PHEX*	LoF	Phosphate-regulating neutral endopeptidase PHEX	Renal phosphate wasting	Hypophosphataemia, X-linked
	193100	AD	*FGF23*	GoF	Fibroblast growth factor 23	Renal phosphate wasting	Hypophosphataemia, AD
	241520	AR	*DMP1*	LoF	Dentin matrix acidic phosphoprotein 1	Renal phosphate wasting	Hypophosphazaemia, ARHR1
	613312 208000	AR	*ENPP1*	LoF	Ectonucleotide pyrophosphatase/phosphodiesterase family member 1	Renal phosphate wasting	Hypophosphataemia, ARHR2, overlap with GACI1
	300554	XL	*CLCL5*	LoF	H(^+^)/Cl(^-^) exchange transporter 5	Renal phosphate wasting	Hypophosphataemia with hypercalciuria, part of Dent’s complex (XLRHR)
	241530	AR	*SCL34A3*	LoF	Sodium-dependent phosphate transport protein 2C	Renal phosphate wasting	Hypophosphataemia with hypercalciuria, (HHRH)
	259775	AR	*FAM20c*	LoF	Extracellular serine/threonine protein kinase FAM20C	Renal phosphate wasting	Raine Syndrome
	309000 300555	XL	*OCLR*	LoF	Inositol polyphosphate 5-phosphatase OCRL	Renal phosphate wasting	Lowe Syndrome Dent-2 disease
	264700	AR	*CYP27B1*	LoF	25-hydroxyvitamin D-1 alpha hydroxylase, mitochondrial	Calcitriol synthesis	VDDR1A
	600081	AR	*CYP2R1*	LoF	Vitamin D 25-hydroxylase	Calcitriol synthesis	VDDR1B
	277440	AR	*VDR*	LoF	Vitamin D3 receptor	Calcitriol receptor	VDDR2A
	600785	AR	*unknown*	LoF	Vitamin D response element-binding protein	Vitamin D response element	VDDR2B
		AD	*CYP3A4*	GoF	Cytochrome P450 3A4	Vitamin D catabolism	VDDR3
	239200	AR	*CaSR*	GoF	Calcium Sensing receptor	Calcium sensing	Neonatal severe hyperparathyroidism
Osteopetrosis & related disorders^a^	259700 604592	AR	*TCIRG1*	LoF	T-cell, immune regulator 1, H+ transporting, lysosomal subunit A3 of V-ATPase pump	Acidification of the resorption lacuna	Severe neonatal or infantile form, and fractures (OPTB1)
	602727 611490	AR	*CLCN7*	LoF	Chloride Channel	Acidification of the resorption lacuna	Severe neonatal or infantile form, and fractures (OPTB4)
	259720 607649	AR	*OSTM1*	LoF	Osteopetrosis associated transmembrane protein 1	β-subunit for CLC-7	Infantile form and fractures, with nervous AR system involvement (OPTB5)
	615085	AR	*SNX10*	LoF	Sorting Nexin 10	Acidification of the resorption lacuna	Severe neonatal or infantile form (OPTB8)
	602642 259710	AR	*TNFSF11*	LoF	Tumour necrosis factor superfamily member 11	Osteoclastogenesis, resorption, survival	Intermediate form, and tendency to fracture (OPTB2)
	603499 612302	AR	*TNFRSF11A*	LoF	Tumour necrosis factor superfamily member 11^b^	Osteoclastogenesis, resorption, survival	Osteoclast poor osteopetrosis, and fractures (OPTB7)
	259710	AR	*CLCN7*	Partial LoF	Chloride Channel	Acidification of the resorption lacuna	Intermediate form, and fractures (OPTA2)
	259700 611497	AR	*PLEKHM1*	LoF	Pleckstrin homology domain containing, family M (with RUN domain) member 1	Vesicular trafficking	Intermediate form (OPTB6)
	259730 611492	AR	*CA2*	LoF	Carbonic anhydrase II	Intracellular acidification	Intermediate form with renal tubular acidosis, and fractures (OPTB3)
	300301	XL	*IKBKG*	LoF	Inhibitor of kappa light polypeptide gene enhancer in B-cells, kinase gamma (NEMO)	Unknown	Osteopetrosis with ectodermal dysplasia and immune defect
	612840	AR	*KIND3/FERMT3*	LoF	Kindlin-3 / Fermitin-3	Cell adhesion	Moderate form with defective leucocyte adhesion (LAD3)
			*CalDAG-GEF1 / RASGRP2*		Calcium and diaclyglycerol-regulated guanine nucleotide exchange factor 1		
	166600	AD	*CLCN7*	Dominant negative effect	Chloride Channel	Acidification of the resorption lacuna	Late-onset osteopetrosis, and fractures (OPTA2, previously known as ADOII)
	265800	AR	*CTSK*	LoF	Cathepsin K	Collagen degradation	Pycnodysostosis, and fractures
**Other Sclerosing / HBM disorders**	155950	AD	*LEMD3/MAN1*	LoF	LEM domain-containing 3	LEMD3 antagonizes the BMP and TGFβ signaling pathways	Osteopoikilosis ^a^
	155950	AD / somatic	*LEMD3/MAN1*	LoF	LEM domain-containing 3		Melorheostosis ^b^
	300373	XL(OSCS)	*WTX*	LoF	Wilms tumour gene on the X chromosome	WNT signaling suppression	Osteopathia striata with cranial stenosis
	224300	AR	*SLC29A3*	LoF	Solute carrier family 29 (nucleoside transporter)	Osteoclast differentiation and function	Dysosteosclerosis
	269500	AR	*SOST*	LoF	Sclerostin	Osteoblast WNT signaling inhibitor	Sclerosteosis
	239100	AR	*SOST*	Reduced function	Sclerostin	Osteoblast WNT signaling inhibitor	Van Buchem’s Disease
	604270	AD & AR	*LRP4*	LoF	Low-density lipoprotein-related receptor 4	Impaired sclerostin-LRP4 interaction	*LRP4* HBM
	603506	AD	*LRP5*	GoF	Low-density lipoprotein-related receptor 5	Osteoblast cell membrane co-receptor regulating WNT signaling	*LRP5* HBM
	awaited	AD	*LRP6*	GoF	Low-density lipoprotein-related receptor 6	Osteoblast cell membrane co-receptor regulating WNT signaling	*LRP6* HBM
	awaited	AD	*SMAD9*	LoF	SMAD family member 9	Inhibits BMP dependent target gene transcription to reduce osteoblast activity	*SMAD9* HBM
	123000	AD	*ANKH*	GoF	Homolog of mouse ANK	Osteoclast-reactive vacuolar proton pump	Cranio-metaphyseal dysplasia
	218400	AR	*GJA1*	LoF	Gap junction protein alph‐1	Osteoclast-reactive vacuolar proton pump	Cranio-metaphyseal dysplasia
	131300	AD	*TGFβ1*	Probable GoF	TGFβ	Cell proliferation, differentiation, migration and apoptosis	Camurati-Engelmann disease
	274180	AR	*TBXAS1*	LoF	Thromboxane synthase	Modulates RANKL and OPG expression	Ghosal haematodiaphyseal syndrome
	151050	SP	*PTDSS1*	GoF	Phosphatidylserine synthase 1	Phospholipid biosynthesis	Lenz‐Majewski hyperostotic dysplasia
	190320	AD	*DLX3*	LoF	Distal‐less homeobox 3	Ectodermal development	Trichodentoosseous dysplasia

GoF, GoF; LoF, Loss-of-Function; ER, Endoplasmic reticulum; UPR, Unfolded Protein Response; COPII, Coat protein complex II; XL, X-linked; VDDR, Vitamin D Dependent Rickets; CN, Cranial Nerve; LAD, Leucocyte adhesion deficiency; ADOII, Autosomal dominant type 2 osteopetrosis

*Where available abbreviations also shown from 2019 Nosology and Classification of Genetic Skeletal Disorders (1).

a When associated with connective tissue naevi, dermatofibrosis lenticularis disseminata then termed Buschke-Ollendorff syndrome

b Asymmetric ‘flowing hyperostosis’ or ‘dripping candle wax’. Approximately 200 cases described to date. Soft tissue changes (hypertrichosis, fibromas, hemangiomas and pain) associated with radiographic features in sclerotome. Contractures can develop.

While not commonly included in bone fragility classifications, we have included pure demineralization disorders caused by a lack of bone mineral supply (genetic forms of rickets/osteomalacia) and osteopetrotic conditions that increase the density of bone mineral and fracture risk, discussed further below (**Table 1**).

### High Bone Mass Disorders

Several rare genetic disorders with skeletal effects, collectively termed osteopetroses and sclerosing bone dysplasias, are associated with a generalized increase in BMD. These disorders can be divided into those in which bone resorption is suppressed, those in which bone formation is enhanced, and those with a disturbed balance between formation and resorption (**Table 1**). Of the currently described 462 genetic disorders of the skeleton, 45 are characterized by osteosclerosis or osteopetrosis, implicating 40 genes (1).

The osteopetroses are rare genetic conditions of reduced osteoclastic bone resorption. Defective bone remodeling induces skeletal sclerosis and abnormally dense, and therefore brittle bones. Osteopetrosis is classified by clinical severity (**Table 1**). Severe neonatal or infantile forms have the worst prognosis (16), while *CLCN7* variants causing osteopetrosis late‐onset form type 2 (OPTA2) (formerly autosomal dominant type II osteopetrosis (ADOII) have a varied clinical phenotype, including asymptomatic forms (17). These mildest forms can be detected as an incidental radiographic finding (18). Pycnodysostosis is caused by defective enzymatic degradation of organic bone matrix, due to autosomal recessive mutations in the gene encoding cathepsin K. Secreted by osteoclasts, cathepsin K cleaves type I collagen (19). The characteristic bone dysplasia includes skull deformities with micrognathia, short stature, dental caries, and abnormally dense, brittle bones (20–22). Understanding of pycnodysostosis prompted development of a novel class of anti-resorptive therapy (e.g*.*, odanacatib) (23).

By contrast, the sclerosing bone dysplasias are associated with increased bone strength and resistance to fracture due to increased bone formation (**Table 1**). Loss-of-function (LoF) *SOST* variants cause sclerosteosis, a rare condition of excessive bone overgrowth (24); a 52-kb deletion located approximately 35-kb downstream of *SOST* is thought responsible for the milder phenotype of van Buchem’s disease (25, 26). Sclerosteosis causes tall stature, mandible enlargement, torus palatinus and mandibularis which complicate tooth extractions (27, 28). Calvarial overgrowth can lead to compression of cranial nerves, particularly facial nerves, sometimes from infancy (27). The underlying *SOST* gene codes for Sclerostin, an inhibitor of WNT signaling that binds to LRP5/LRP6 co-receptors to decrease osteoblastic bone formation. LoF *SOST* mutations result in increased osteoblastic bone formation. Similarly, variants in *LRP5* and *LRP6* which prevent sclerostin binding can lead to HBM (29, 30). More recently a novel, likely LoF variant in the DNA-binding domain of *SMAD9* was reported in three kindreds with HBM, displaying features of mandible enlargement, torus palatinus and torus mandibularis (31). Unusually, a feature which seems to be common to these sclerosing bone dysplasias is an inability to float!

## Careful Patient Phenotyping Is Key to Disease Discovery

It is evident that the spectrum of abnormal skeletal phenotypes is wide, highlighting the importance of a thorough phenotypic evaluation to determine disease characteristics and the extent of skeletal and extra-skeletal manifestations in patients with a suspected rare bone disease. A detailed patient history is essential and should provide information regarding growth and development, previous and concurrent illnesses including any medical treatment, dental health, skeletal and non-skeletal symptoms, physical activity and potential restricted mobility, and other lifestyle factors. Family history is important, both in adults and children. A fracture history should cover all fractures sustained from childhood to present age, and must be accompanied by age at fracture, fracture site, fracture mechanism (low, moderate or high-energy trauma), treatment, and fracture healing duration (1).

Careful phenotyping includes routine physical examination, with attention for the existence of extra skeletal signs of OI such as blue sclerae, dental abnormalities, hyperlaxity of skin and joints, and signs of secondary diseases like Cushing syndrome, thyroid disease or malnutrition. Laboratory investigations should include relevant parameters to determine calcium-phosphate homeostasis and to exclude secondary causes of osteoporosis. The extent of tests depends on the patient’s age and clinical manifestations, and can include e.g., serum calcium, phosphate, alkaline phosphatase, 25-hydroxyvitamin D, parathyroid hormone, thyroid function, gonadal hormones, prolactin, glucose, complete blood count, erythrocyte sedimentation rate, C-reactive protein, kidney and liver function, ferritin, celiac serology, urinary calcium excretion, and bone turnover markers. Further analyses should include appropriate radiological investigations (plain radiography, CT, MRI, radionuclide bone scintigraphy), and bone density assessment by DXA (**Table 2**). In selected cases, a transiliac bone biopsy may help in diagnosis but requires analysis by an experienced histopathologist with very specific bone expertise. Repeat measurements of BMD may indicate whether low bone mass is due to inadequate bone accrual or increased bone loss.

**Table 2:** Tools and relevant clinical outcomes to be considered for careful skeletal phenotyping in patients with a skeletal phenotype.

Tool	Phenotypic information
**Clinical evaluation**	Age at onset? Abnormal height and weight?
	Abnormal body proportions?
	Dysmorphic features?
	Deformities?
	Asymmetry?
	Dental or oral abnormalities?
	Neurological manifestations?
	Hearing and/or visual loss?* Pain (back, bone)? Muscle weakness? Cutaneous lesions (e.g., fibrous dysplasia, mosaic RASopathies)? History or signs of systemic conditions influencing bone? Other extra-skeletal manifestations?
**Biochemistry**	Abnormal bone mineral homeostasis (calcium, phosphate, alkaline phosphatase, Vitamin D, Parathyroid hormone? Abnormal bone turnover?
	Endocrine or renal disturbances?
	Signs of secondary skeletal fragility (e.g., celiac disease, chronic inflammation)
**Radiography**	Abnormal bone texture (e.g., fibrous dysplasia, sclerosis)?
	Abnormal bone modeling?
	Evidence for skeletal dysplasia?
	Spinal compression fractures?
	Scoliosis, other spinal deformity?
	Abnormal cortical/calvarial thickness?
	Skeletal deformities?
	Abnormal bone maturation (in children)? Signs of nerve compression?
**Bone density**	
** DXA**	Abnormal bone mineral content and BMD?
	Vertebral fractures (lateral spine, possible with some DXA machines)?
** pQCT & HRpQCT**	Bone characteristics at peripheral sites (in research settings)?
** QCT**	Bone characteristics of vertebrae (in research settings)?
**Transiliac bone biopsy (double labeled)**	Abnormal bone histomorphometry (structure)? Abnormal mineralization density (BMDD)? Abnormal bone cell proportions?

*Consider formal audiometry and visual field assessment.

In a clinical setting, the means to obtain detailed information about the bone tissue characteristics are usually limited. However, if a novel skeletal phenotype with low or high bone mass is suspected, several research tools can be used to determine the bone characteristics in peripheral (pQCT, HRpQCT) or central (QCT) sites. Furthermore, a double-labelled, transiliac bone biopsy can be analysed using multiple techniques. The standard histological and histomorphometric evaluations can be complemented with back-scattering electron imaging, Raman microspectroscopy and even immunohistochemistry. In addition, a transiliac bone biopsy provides the opportunity to obtain a bone marrow sample for mesenchymal stem cell (MSC) and osteoblast cultures. In case of HBM disorder it can be hard to obtain a bone biopsy. Skin fibroblasts, retrieved from the affected individual either at the bone biopsy site or, more commonly, from the forearm skin, are also widely used in various functional studies.

Altogether, rigorous phenotyping remains an imperative step in disease discovery and interpretation of disease causation. To this effect, GEMSTONE has recognized the importance of careful phenotyping, setting up **Working Group 2** (‘**Phenotyping**’) aimed at disentangling the complex heterogenous components of monogenic and polygenic bone disease into more well-defined processes. A detailed review of this is described elsewhere (32).

## Genetic Approaches to Gene Discoveries

**Figure 1:**
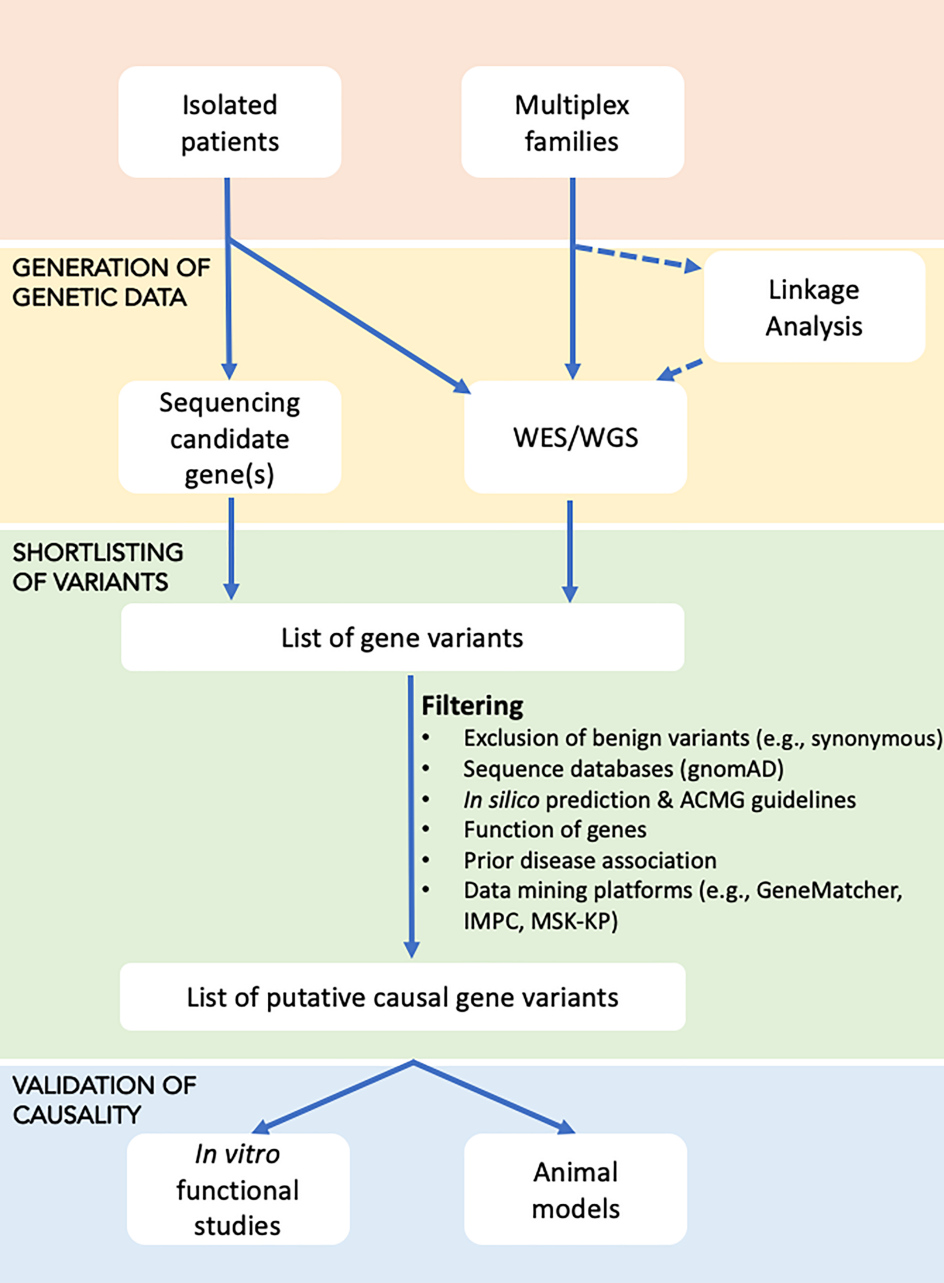
Flowchart for identifying the underlying genetic cause in disorders with increased or decreased bone mass. Affected individuals, either as isolated cases or as part of a family study, are subjected to genetic testing in the form of targeted sequencing (using Sanger or high-throughput sequencing of candidate genes), or WES/WGS, with the latter possibly undergoing prior linkage analysis to define loci that are shared by affected relatives and thus likely to harbour the causal gene(s). Sequential filtering steps are applied to narrow down the extensive list of gene variants to a few variants that based on database and literature searches are most likely to explain the disease. Shortlisted gene variants are functionally validated using *in vitro* cell work or *in vivo* animal models.

In individuals with a suspected monogenic disorder of increased or decreased bone mass, the approach presented in the flowchart could be used to identify the genetic cause (**Figure 1**).

If the clinical diagnosis is clear, the genetic analysis can be restricted to one gene or a limited number of genes. Depending on the size and the number of genes, Sanger sequencing may be appropriate. However, in most cases high-throughput sequencing using a gene panel will be more cost- and labor-efficient. For the analysis of genes involved in OI, several gene panels are commercially available. For cases with increased bone mass, availability is more limited but often the relevant genes are included in wider skeletal dysplasia gene panels. In addition, academic groups have developed gene panels for research purposes.

Since the number of gene panels is ever increasing and patients with less severe phenotypes are subjected to genetic investigations, clinicians often face difficulties interpreting genetic results. A general and reasonable recommendation is to follow the American College of Medical Genetics and Genomics (ACMG) guidelines (33) and only report those variants found to be either ‘likely pathogenic’ or ‘pathogenic’ (ACMG scores 4 and 5, respectively). For variants of unknown significance (ACMG = 3) the clinical significance remains uncertain and further work may be required to confirm or refute pathogenicity of the variant. Sometimes, analyses of family members can help to identify whether a variant segregates with the phenotype. In research settings, it would be optimal to set up a “rapid throughput screening pipeline” to help prioritize the most interesting potentially causal variants, which might be carried forward for functional analyses.

A negative genetic screening result might indicate the involvement of a currently unknown disease gene, as such genes are obviously not included in established gene panels. An exome-wide analysis starting from data generated by whole exome sequencing (WES) or whole genome sequencing (WGS), assessing all genes in the human genome, may offer an opportunity to identify these novel genes and/or gene variants. However, this approach, as well as the use of extended gene panels, will result in the identification of a large number of gene variants, necessitating variant filtering and variant prioritization in order to end up with a short list of putative causal variants.

A plethora of filtering steps can be used for WES and WGS data analyses. First degree relatives share 50% of their genome. Thus, inclusion of other affected and non-affected relatives from an extended family can help eliminate the shared benign genetic variation shifting the focus on causal gene variants. This is a pertinent step when dealing with the wealth of data generated from high-throughput sequencing. Contrary to the family-based approach, incorporation of unrelated patients sharing the phenotype should be done with caution. The genetic basis of phenotypically similar monogenic conditions can be heterogeneous, and assuming a shared disease-causing gene variant in all similarly affected individuals may well result in the exclusion of the true causative variant.

**Figure 2:**
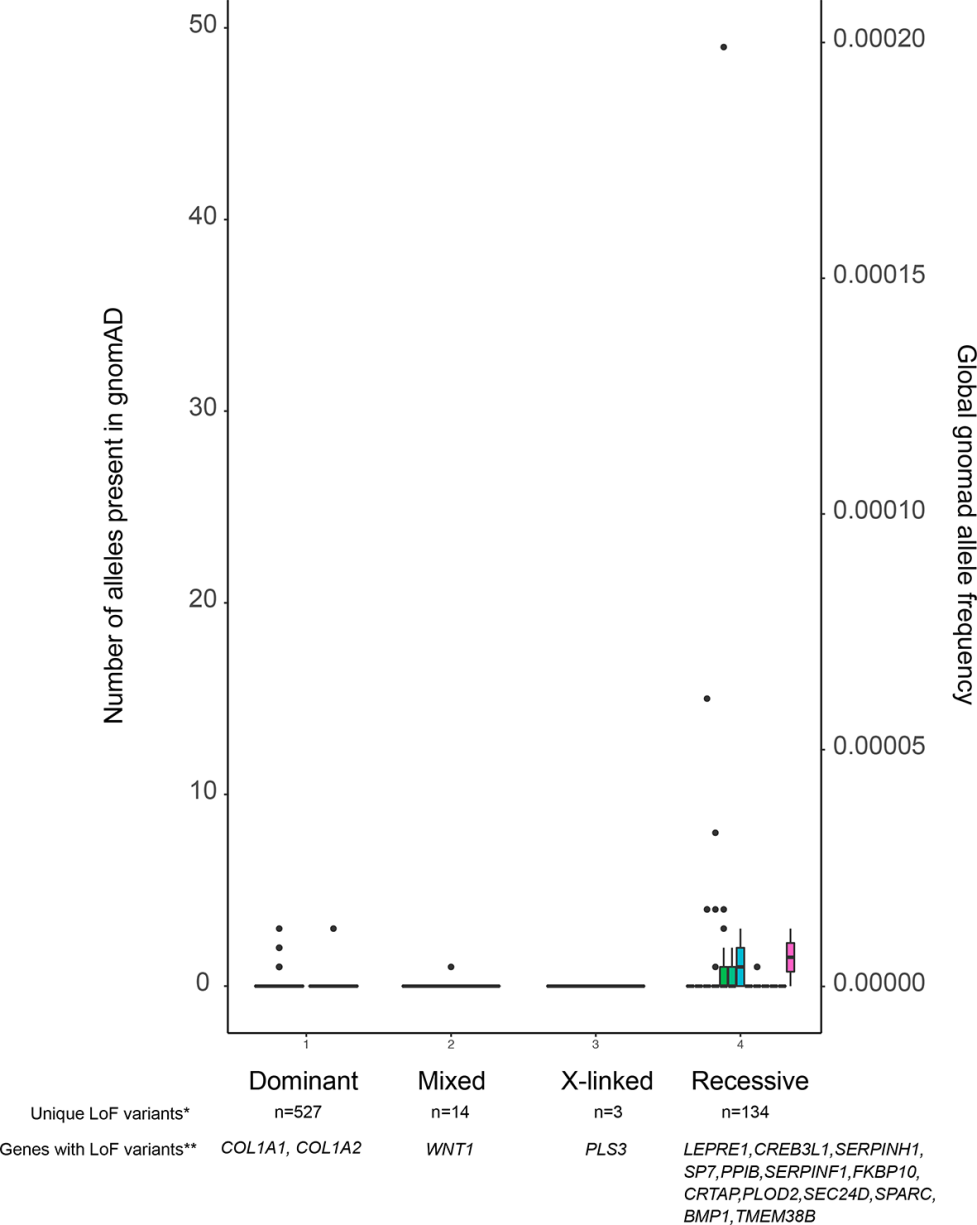
Allele frequencies of LoF variants in the OI database. The figure displays all variants, and their allele frequencies, reported to the OI database which is contained within the Leiden Open Variation Database (LOVD). Because the LOVD also contains benign variants, we have chosen to use LoF variants as a proxy for all pathogenic variants. As is shown, pathogenic variants are not only very rare in dominant OI genes, but they are also very rarely found in recessive OI genes. *Number of unique LoF variants in OI genes reported in the LOVD. **Genes with reported LoF variants in LOVD. Note that not all known OI genes have LoF variants, as annotated by VEP (35), reported in LOVD.

The presence of variants in adult population databases such as gnomAD (34) with a frequency above a selected threshold can serve as a criterion for exclusion of these variants. According to ACMG guidelines, an allele frequency above 5% can be used directly to dismiss a variant as disease-causing. Furthermore, as shown in **Figure 2**, reducing this threshold to 0.1% is acceptable, as it is unlikely to result in a high risk of missing the relevant variant, especially in autosomal dominant diseases. Short insertions or deletions (indels) need special attention, because although they are often predicted to cause LoF, the variant calling algorithms are not as reliable as for single nucleotide variants (SNVs) (36). For further selection, functional predictions for the variants and/or current knowledge regarding gene function must be taken into account. Many bioinformatics tools are available for pathogenicity prediction of variants, such as Sorting Intolerant From Tolerant, SIFT [(37) (https://sift.bii.a-star.edu.sg/), Polymorphism Phenotyping v2, PolyPhen-2 (38) (http://genetics.bwh.harvard.edu/pph2/], Combined Annotation Dependent Depletion, CADD (39) (https://cadd.gs.washington.edu/), and Variant Effect Predictor, VEP (35) (https://useast.ensembl.org/Homo_sapiens/Tools/VEP), and for linking the variants with disorders by matching the clinical phenotypes as defined by HPO (human phenotype ontology) terms (40). In order to check whether other variants in the same gene have been found somewhere in the scientific community, data can be uploaded to GeneMatcher (41) (https://genematcher.org). When assessing the relevance of genes in a candidate variant list, one can integrate information from several resources. Some examples are the Musculoskeletal Knowledge Portal (42) (http://mskkp.org/), which provides a search engine for the results of genome-wide association studies (GWAS) on bone phenotypes, and the International Mouse Phenotyping consortium, IMPC (43) (www.mousephenotype.org) to consult bone phenotypes of knock-out mice. As the number of new gene discoveries increases rapidly in low and high bone mass disorders, a thorough literature review should also be a part of the assessment process. Filtering of variants to only one or very few potentially disease-causing variants should be first validated by Sanger sequencing or other genotyping methods, followed by appropriate functional studies, *in vitro* or *in vivo*, to validate the disease causality of the selected variant.

Compared to the genome-wide approach, WES is currently a more money and time saving option in terms of data storage and data analysis. However, as the costs of DNA sequencing are rapidly decreasing, WGS is becoming increasingly affordable, even within a clinical setting. One can use the obtained data as a virtual WES analysis by only looking at the coding parts of the genome. A very important advantage of WGS is that all genes are in general completely covered with relatively even coverage, unlike WES which requires an additional step of DNA capture that results in incomplete and uneven coverage of some genes. Furthermore, WGS allows for structural variant analysis (44) and opens up possibilities to gain insights into the involvement of non-coding parts of the human genome.

As described, filtering of identified variants by WES is already a difficult process which becomes even more challenging when using WGS data. The number of variants increases exponentially and the development of tools for validation of variants in non-coding areas is currently one of the most challenging aspects of genetic research. Copy number variant (CNV) detection, although possible from WGS data, is limited with the current short-read technology. However, long-read massive sequencing such as that provided by PacBio or Oxford Nanopore, may become available for clinical applications in the near future. Germline variants in microRNAs and long non-coding RNAs should also be kept in mind as they have been reported as an underlying cause in a few skeletal disorders (45).

If no variant is identified by WES or WGS that can reasonably explain the patient’s high or low bone mass phenotype, it may be worth exploring patterns of gene expression using RNAseq approaches. In this way, splice variants far from the canonical splice-site can be detected, as well as monoallelic expression. Also, total absence of a given transcript or appearance of a new, potentially rare, alternative transcript unique to the patient’s RNA and absent in control samples, provides strong evidence for the involvement of the gene in disease pathogenesis. Ideally, one would choose a single-cell approach and access bone cells to obtain total RNA, which is understandably, not always feasible. Instead, proxy tissues must often be used, such as lymphocytes or skin fibroblasts. RNAseq is becoming quite popular as a second line tool for gene identification and some nice examples of new pseudo-exons due to deep intronic variants have been identified in this way (44). However, methods are relatively expensive, technically demanding and require specialized analysis.

Finally, a note about GWAS. It is important to understand that in general such studies do not serve as tools for gene discovery in monogenic diseases. GWAS performed at the population level using large cohorts, have been very powerful in defining the polygenic basis of BMD. They have been crucial in highlighting pathways and genes involved in the regulation of bone mass and fracture susceptibility. In many instances, the same gene, with rare penetrant variants causing a monogenic BMD phenotype, has been associated with common variants conferring susceptibility for the polygenic inheritance of BMD (7, 46). The paradigm of this is *LRP5*, a gene responsible for several monogenic conditions including HBM and osteoporosis-pseudoglioma syndrome, but also the locus of genome-wide-significant SNVs strongly associated with hip and spine BMD (47, 48). Nevertheless, this does not apply to all genes underlying monogenic skeletal dysplasias. GWAS results may soon lead to the development of a tool to calculate a polygenic risk score (PRS) for any given individual, allowing for prediction of the risk for abnormal bone mass and possibly fracture risk, based on an individual’s combination of susceptibility variants (49). While this is still not a clinically available application, there will probably be a time when we can combine PRS and rare variant scrutiny through WES or WGS to gain precision in genetic diagnosis of high or low bone mass phenotypes.

## Validation of Genetic Findings

### Identifying the Best *In Vitro* Model

A fast and simple approach to functionally validate novel candidate SNVs is through cell-based *in vitro*/*ex vivo* studies. Experiments should be designed according to the type of identified gene variant, the disease inheritance model, gene expression levels and the associated protein function. Use of appropriate cell lines is fundamental in recapitulating the observed phenotype seen in affected individuals. Primary bone cells harbor numerous advantages since they provide insight into the function of previously unknown genes and can also be used to investigate the defective cells’ function in comparison to that exhibited by otherwise ‘healthy’ cells. For instance, primary osteoblast cultures retrieved from a type VI OI patient with a novel *de novo* dominant *IFITM5* mutation, revealed that the defective *IFITM5* resulted in a reduced *SERPINF1* expression during cell differentiation, decreased type I collagen expression and secretion, and induced mineralization defects. Type VI OI is typically caused by recessive null mutations in *SERPINF1.* However, findings supported the relationship between the two genes and highlighted their role in different OI types and bone mineralization (50). Moreover, rescue experiments have proved to be an effective tool in recovering protein function, complementing the results obtained in gene manipulation studies (51).

Unfortunately, access to bone biopsies remains a challenge and further complicates the limited availability of abnormal tissue. To overcome this difficulty, induced pluripotent stem cells (iPSCs) derived from patient fibroblasts can be reprogrammed to differentiate into bone cells, reproducing the disease model of interest (52–54). Moreover, if patient samples are unavailable, cell lines modified by genetic engineering techniques (such as CRISPR/Cas9) can be applied for validation studies. Immortalized primary cells (from humans or mice) such as osteoblasts (bone-forming cells e.g., hFOB), mechanosensing osteocytes (e.g., ocy454, MLO-Y4), osteoclasts (bone-resorbing cells e.g., RAW264.7), bone marrow-derived MSCs (precursors of osteoblasts); and cancer-derived (i.e., osteosarcoma cells [e.g., SaOS2, MG-63], or osteolytic [e.g., PC-3]) cell lines or adipose tissue-derived stem cells can be utilized. Next, it is necessary to simulate the pathological phenotype by generating or introducing the genetic abnormality in the selected cells. The best way to achieve this is to disrupt gene function and analyze cell function. It is crucial to assess the steady state levels of the protein encoded by the mutated gene, as well as the effect of the variant on protein function. Aberrant protein function, possibly complete ablation, is expected for LoF and missense variants. Researchers can experimentally reduce gene expression at the translational level using RNAi (knockdown) (55) or interrogate gene function by completely and permanently silencing it through CRISPR/Cas9 genome editing (knockout) (56). Alternatively, identified gene variants (such as SNVs, and indels) can be introduced in plasmids expressing the gene of interest using site-directed mutagenesis (SDM) or CRISPR-directed gene editing, followed by transfection in specific cell types. Indeed, Collet et al. (2018) demonstrated the pathogenicity of several *LRP5* missense variants following SDM that led to diminished luciferase activity due to reduced activation of canonical WNT signaling (57). Similarly, different *PHEX* variants identified in X-linked hypophosphatemic rickets, both splicing and *de novo* mutations, were introduced in minigene constructs by SDM and functionally confirmed by gene expression profiling (58) and evaluation of protein stability (59).

Finally, once cells are genetically modified, several tests may be applied according to the expected pathogenic phenotype in the evaluated cell type. For example, mineralization capacity may be evaluated in osteoblasts by means of Alizarin Red S staining of calcium precipitates. Alternatively, pit assays and tartrate-resistant acid phosphatase (TRAcP), together with actin ring staining can be used for exploring osteoclast differentiation and function. Bone cell-specific markers may also be measured at the messenger RNA level (using quantitative real-time PCR) or at protein level (by ELISA or Western blot) (60). As an illustration, bi-allelic variants in the tissue non-specific alkaline phosphatase (*ALPL*) gene, involved in the hypophosphatasia (HPP) phenotype, were expressed in human osteosarcoma (U2OS) cells and assessed by measuring ALP activity, as well as the mineral deposition using Alizarin Red S staining. Results demonstrated that the location of the variant in the ALP enzyme structure correlated with the severity of the phenotype (61). Another example is the comprehensive study of differentiated osteoclasts from *Pls3* knockout and *Pls3* overexpressing mouse models. *PLS3* variants were found to influence bone resorption in patients with X-linked early onset low-turnover osteoporosis (62). Osteoclasts lacking or overexpressing *PLS3* were evaluated using several techniques, including TRAcP staining and resorption assay; protein expression analysis by immunohistochemistry and western blotting; identification of PLS3-binding partners by mass spectrometry and co-immunoprecipitation; and gene expression profiling. Finally, authors concluded that unbalanced PLS3 levels affected osteoclast development and function by dysregulation of the NFκB pathway (62).

The current limitation of *in vitro* cell studies is the difficulty in predicting or interpreting the *in vivo* behavior since cells lack critical cell-tissue interactions, mechanical stimuli and paracrine factors that are pertinent in understanding the complexity of bone physiology. Hence, cell-based results should be interpreted with caution when extrapolated to bone as a whole organ.

### Mouse Models: The ‘Gold’ Standard for Functional Validation

One of the biggest challenges in the validation of a specific SNV as the cause of a monogenic disease is how to model the genetic condition. Although there are different strategies for validating SNVs, the use of a mouse model remains the current gold standard. Indeed, there are many key advantages and characteristics favoring this model for the investigation of heritable conditions, whether they are rare or common. These include the high degree of genetic and mechanistic conservation between mice and humans, the high fecundity, and the short life cycle and gestation period (63). Furthermore, mouse models allow the consequence of the genetic abnormality to be determined during intrauterine development, postnatal growth, adulthood and ageing and following appropriate provocation. Nevertheless, limitations concerning the use of mouse models with respect to humans exist. Indeed, some human diseases are not easily modelled in mice, most of the mouse models used in research are inbred and do not capture the genetic variation that exists in humans, and early mutant phenotypes are difficult to study. Moreover, other limitations of this animal model are represented by the differences between mouse and human skeletal structures and dynamics, lack of standardized protocols for analysis, difficulty of *in vivo* cell imaging, as well as statistical and ethical issues (64).

Over the years, genetic bone diseases have been studied by applying reverse and forward genetic approaches in the mouse model (65). An important example is represented by mouse models carrying variants in the *Wnt1* (66) and *Pls3* (62), recapitulating low bone mass syndromes according to the underlying genetic variant.

Before proceeding with the selection or generation of a mouse model, a primary consideration should be made regarding the nature of the SNV to be investigated. Firstly, cross-species gene conservation should be determined, along with the mode of inheritance of the disease (67). This represents a crucial step in establishing the design strategy or choice of animal model. Currently, different methods exist to generate a mouse model, including gene targeting embryonic stem (ES) cells, conditional mutagenesis, pronuclear injection-based transgenesis and CRISPR/Cas9-based genome editing (68). Whichever strategy is used, the model generated must have a measurable phenotype that recapitulates the human disease in terms of pathological characteristics and disease progression.

Once the mouse model has been generated and validated, the resulting bone phenotype must be assessed, possibly at different levels. One of the most commonly used methods to investigate the presence of bone alterations in small rodents is microcomputed tomography (µCT), which provides information on bone mass, density and 3D microarchitecture (69). To date, µCT allows both *ex vivo* and *in vivo* bone analysis. A quicker phenotyping approach is through classical 2D X-rays. Unlike µCT, X-ray analysis has low resolution and information is limited to the presence of gross skeletal abnormalities (69). Furthermore, *ex vivo* tissue retrieved from mice can be used to model bone fragility and bone quality (46) by indentation or three-point bend and compression testing.

Another essential tool for the investigation of the bone phenotype at the cellular level is histomorphometry (70). Serological analysis of several bone formation and resorption markers can also be performed, including N-Terminal Propeptide (PINP), bone ALP (bALP), TRAcP, N-terminal telopeptide (NTx), and C-terminal telopeptide (CTx) (71).

Finally, the mouse model may represent a source of primary bone cells. This aspect is very important especially if primary cells from patients are not available due to technical or ethical issues (72, 73). Overall, the mouse model remains an essential tool for modeling monogenic bone disorders. The techniques, benefits, limitations, costs and applications of different translational model systems are described in more detail in another review by **GEMSTONE Working Group 4** entitled ‘Functional Investigations’ (74).

### Zebrafish Are an Attractive Model System for Novel Gene Discoveries

A major bottleneck in translating findings from clinics to lab benches is the need for a fast and affordable process to test gene function and drug effects. Zebrafish (*Danio rerio*) are a small tropical fish that are highly fecund, have a fully annotated genome (75) and are relatively cheap to maintain since they are kept in large shoals. As the vertebrate skeleton is largely conserved over evolution, zebrafish have osteoblasts, osteocytes, and osteoclasts (both mono and multi-nucleated) (76) that are tightly coupled with bone metabolism regulated by similar pathways (WNT, BMP, parathyroid hormone, etc.) as described in humans (77). Traditionally, zebrafish have been a popular model organism in developmental biology as the translucent embryos are laid extra-maternally making them very accessible for *in vivo* imaging experiments. More importantly, they are easy to manipulate genetically and have been used for forward genetic screening, gene knockdown (by micro-injecting antisense RNA morpholinos), or reverse genetic approaches using targeted endonucleases (gene editing) micro-injected into one-cell stage zygotes (78, 79). The embryos develop rapidly; the first cartilage structures appear at 2 days post fertilization (dpf) in the craniofacial area, whereas dermal bone elements form as early as 3 dpf. Endochondral ossification occurs as early as 5 dpf and all these processes can be imaged *in vivo* and tracked over time. The notochord initiates its mineralization process as early as 4 dpf and acts as a template to give rise to the spinal cord structures comprising of vertebrae and intervertebral discs (80).

**Figure 3:**
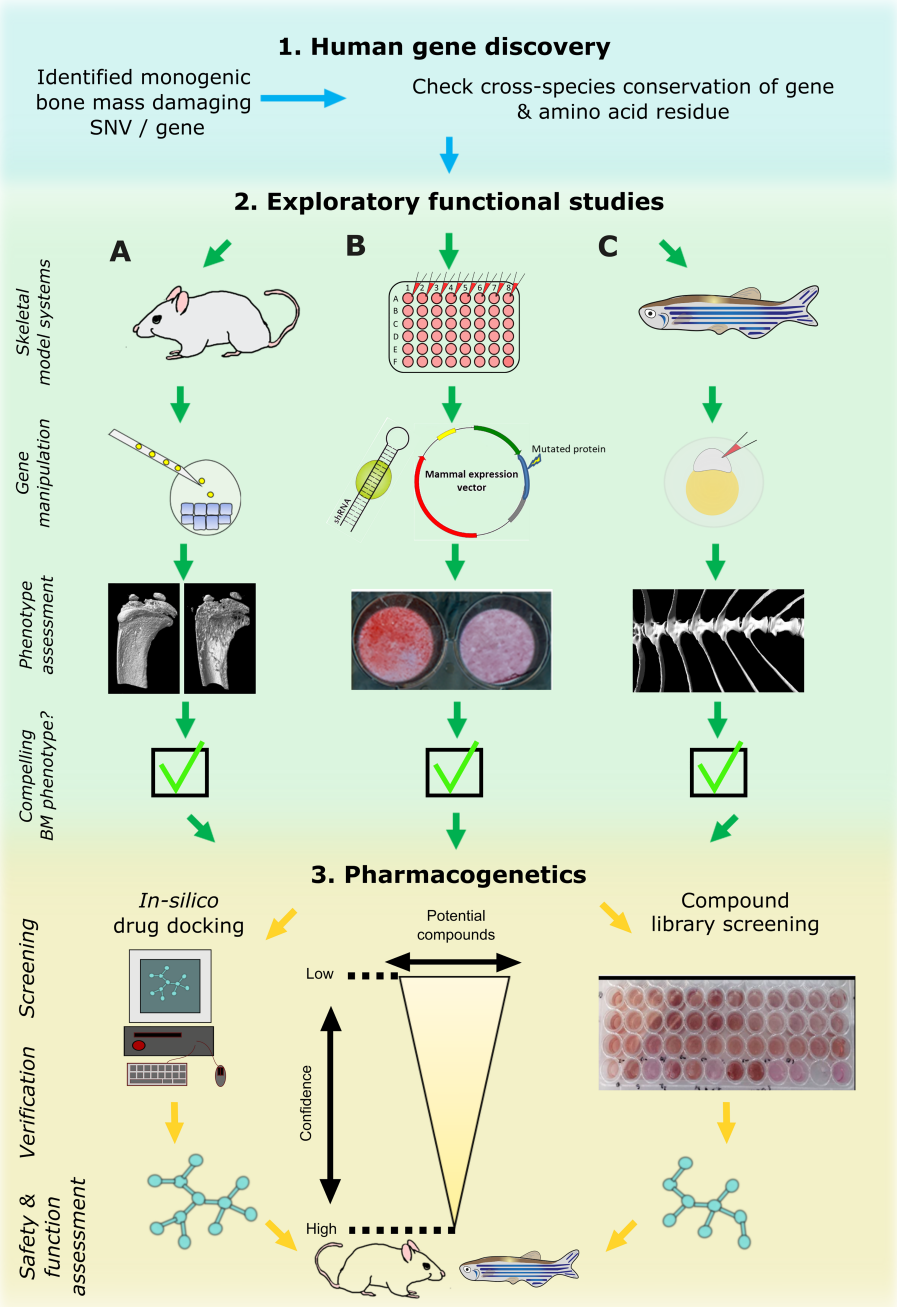
Cross laboratory collaboration to perform functional validation in multiple model systems following a novel gene and variant discovery. Schematic representation of proposed pipeline to functionally validate a newly identified damaging mutation in a gene. First, evolutionary conservation of the gene and ideally of the variant should be checked to determine downstream study strategies in the three main model systems relevant to bone: mouse **(A)**, tissue culture cells **(B)**, and zebrafish **(C)**. These model systems are often utilized sequentially but their use in parallel is likely to enhance output. The main focus of primary functional studies are gene manipulation (knockdown or knockout) of the whole gene which generate read outs relevant to bone (i.e. CT or mineralization assays). If all three model systems provide compelling data, the pharmacogenetics phase could provide first data of potential ways to target the new protein by performing high performance computing *in silico* drug docking strategies and/or screen compound libraries in multi-well settings (mainly *in vitro* cultured cells and/or zebrafish *ex vivo* cultured scales). Newly identified ‘high confidence’ compounds could then be assessed in adult mice and/or zebrafish for further validation.

With the recent advent of fast CRISPR/Cas9 protocols and in combination with the widely available transgenic reporter lines that drive fluorescent proteins under control of cell-type specific promoters (such as *sp7(osx):GFP* [osteoblasts] and *col2a1a:mCherry* [chondrocytes]), it is feasible to rapidly test gene function related to skeletal dysplasias or early mineralization defects in zebrafish larvae (77, 81, 82) (**Figure 3**). Furthermore, it is possible to ablate specific cell types, such as osteoblasts, chemically by fusing nitroreductase (83) with a fluorescent protein which is driven by a cell-type specific promoter (e.g. *sp7(osx):mCherry-NTR*). This allows temporal control of the ablation by simply adding NTR to its substrate Nifurpirinol (antibiotic) in water (84). These and other genetic tools are valuable to follow *de novo* osteoblastogenesis and bone formation in a mutant fish or in a pharmacological setting.

Only in the past few years have the adult skeletal ageing phenotypes been more thoroughly explored. Adult zebrafish have extensive endoskeletal (e.g., vertebrae, dermal skull elements, endochondral jaw elements) and exoskeletal (e.g., scales, fin rays, teeth of dermal origin) structures that can be assessed (80). As the zebrafish skeleton is fully mineralized by 3 months of age, the development of computed tomography (33) techniques for small samples (µCT) allow detailed quantification of shape, calcium content and bone volume of the adult zebrafish skeleton (82, 85). Just as in mammals, the zebrafish bone architecture responds to glucocorticoids, and loading related to physical exercise and diet (e.g., high fat, high glucose, ferric ammonium citrate and high calcitriol) (86–90). Moreover, the zebrafish exoskeleton (scales and fin rays harboring osteoblasts and osteoclasts) is easily accessible for experimental procedures and exhibit the same osteoanabolic gene expression profile (91), providing a source of bone remodeling tissue that can be used for bone injury assays (92, 93) and *ex vivo* multi-well culture experiments (94).

As demonstrated with the *chihuahua* (*col1a1a*) mutant, an OI model identified during a forward genetic screen, *chihuahua* mutant fish have increased mineralized matrix but have brittle bones that remarkably recapitulate the phenotype exhibited in affected humans (95). More detailed description of additional mutants (such as *sp7, bmp1a, plod2, entpd5a, mmp9*) as bone disease models have been discussed in recent reviews (77, 82, 85, 96). Since adult fish are kept in large shoals, it is possible to screen libraries of mutants for specific bone phenotypes using µCT and then correlate that to the genotype using high-throughput sequencing approaches (97). Reverse genetic protocols have improved with CRISPR/Cas9, as specific point mutations can be introduced in the zebrafish genome (98, 99). This allows the study of specific genes and gene variants implicated in HBM, LBM, and other skeletal dysplasia in zebrafish skeletal models. Hence, combining the power of genetics and the relative ease of pharmacological manipulation (*in vivo* and *ex vivo*), make zebrafish are an attractive model to study novel genetic factors discovered in humans.

## Using Complementary Strengths of *in Vitro* and *In Vivo* Models to Accelerate Pharmacological Discoveries

Increasing knowledge of genetic factors involved in bone metabolism, particularly mechanisms controlling gene regulation, cell signaling and differentiation, has inspired the design of drugs to directly target the underlying defect by inhibiting osteoclastic bone resorption and/or promoting osteoblastic bone formation. The goal is to identify small molecular weight compounds that selectively activate or repress expression of key genes or target specific histone marks that subsequently alter the corresponding protein level and signaling pathway. This can be achieved using cells and animal models as discussed below. Alternatively, the availability of the ever-growing, highly dimensional omics data that is tissue- and species-specific has necessitated the need for computational methods using computer-aided drug design to analyze gene expression profiles for drug target predictions (100).

An initial mandatory step in drug identification and preclinical testing of a novel pharmacological compound involves the use of cells, ranging from *in vitro* cell cultures (primary cells, cell lines or iPCS), *ex vivo* bone explants, and the more recent 3D cell culture models, to determine the beneficial and toxic effects of candidate drugs. Additionally, banked stem cells with a wide variety of genetic backgrounds enable a more thorough interrogation of the therapeutic options increasing the possibility of identifying novel drug targets. Overall, the major challenge of an effective treatment is to rescue the “healthy” phenotype and, if possible, keep this improvement permanent.

Studies have also demonstrated the utility of cells in relation to drug dosing. Li et al. utilized induced primary osteoclasts to identify the minimal effective concentration exerted by zoledronic acid for osteoclast suppression with sufficient alteration in cell adhesion, migration and resorption (101). In a second example, low doses of odanacatib induced higher anti-resorptive activity in cultured osteoclasts compared to those treated with a 100-fold higher dose of the same drug. Exposure triggered the stimulation of other cysteine proteinases culminating in increased collagen degradation mimicking that observed in patients (102).

Despite their limited costs and quick turnaround time, *in vitro* cell experiments suffer from several pitfalls in that they cannot fully capture the complex drug effect and interactions manifested *in vivo*. Hence, results obtained in a culture plate should be interpreted with caution.

The use of mammalian models represents a “mandatory” step in pharmacological discovery, both prior to or following gene identification. Animal models present a more holistic and unbiased set-up for the investigation of known or novel bone bioactive drug compounds. *In vivo* compound testing across different model systems can be performed with varying degrees of success. Zebrafish are increasingly used for *in vivo* drug discovery, drug repurposing, and chemical risk assessment. They bridge the gap between *in vitro* cellular studies and *in vivo* mouse models, and in contrast to mouse models, can be scaled up for high-throughput drug screening. This is because zebrafish embryos/larvae (WT or genetically altered) can be arrayed in 96-well plates exposed to small molecules dissolved in their water and several replicates can be tested concurrently increasing statistical power. Additionally, assays are of shorter duration. Thanks to a rapidly developing skeletal system, transgenic lines (e.g., *sp7(osx)*, *oc2*, *runx2a*, *col2a1a*) can be tested for specific skeletal elements and cells, circumventing the need for sacrificing. In addition, only small drug quantities are required unlike other mammalian dosing studies (103). A successful example of an osteogenic drug identified in a zebrafish chemical screen is Dorsomorphin, a SMAD-dependent BMP type-II receptor signaling inhibitor specifically named after the striking phenotypic dorsalization effect in zebrafish embryos (104), having therapeutic potential to treat fibrodysplasia ossificans progressive (FOP). Additionally, the larval superficially located operculum and adult zebrafish scales and caudal fin (before and after removal) can be used to study the drug’s effect on osteogenesis. Indeed, prednisolone-treated zebrafish scales (containing osteoblasts and osteoclasts in their native environment) reproduces the same glucocorticoid-driven osteoporotic phenotype as observed in humans (105); the phenotype is rescued with alendronate (106). Moreover, scales can be harvested from adult fish and then cultured *ex vivo* in a 96-well setting which allows testing of various compounds simultaneously without exposing the individual fish to potentially harmful pharmacological agents (77, 94).

Zebrafish mutants modelled on rare human bone disorders, such as OI, can also provide new avenues for drug discovery. An improvement in the degree of bone mineralization was observed in *chihuahua* mutant larvae treated with the chemical chaperones 4PBA and TUDCA (107), whereas the *frilly fins* mutant (defective *bmp1a*) responded to anti-resorptive therapy, similar to OI patients with *BMP1* mutations (93, 108). In summary, zebrafish provide a versatile platform for drug discovery in bone disorders, with pharmacological effects seen in zebrafish and humans being highly conserved. Thus, any promising hits are more likely to succeed and can be taken to higher animal models prior to clinical trials (109).

Rodents, such as mice and rats, have been widely used for drug testing in common and rare bone diseases. A classic example is represented by ovariectomized mice or rats, which have been used to test anti-resorptive (e.g., bisphosphonates, odanacatib) and anabolic drugs (e.g., teriparatide, anti-sclerostin monoclonal antibody [Scl-Ab]) (110, 111). Moreover, the use of transgenic mouse models has also allowed drug discovery studies to be extended to rare bone diseases facilitating the development of targeted therapies (112).

The versatility of mouse models has enabled pre-clinical human studies to progress toward drug development. The effectiveness and specificity of the candidate compound, together with the biodistribution within tissues, as well as clearance and safety can be tested in mice (113). Drugs can be administered through different routes – systemic or local (114). Moreover, mice are suitable for testing different types of drugs, including small molecules, antibodies and RNA interference (siRNA, shRNA and miRNA).

There are however limitations hindering the use of mice for drug discovery. The main pitfalls remain the limited number of compounds that can be tested due to the expenses related to mouse purchasing and housing, the quantity of drugs used, and post-treatment analyses (113). Overall, these represent essential features for translating a potential therapy in the clinical setting to treat patients. Importantly, most of the discussed aspects can only be partially tested in other experimental models, including *in vitro* and non-vertebrate models, thus making the mouse an indispensable tool in the drug discovery pipeline.

Another crucial aspect to be considered when using *in vivo* animal models in drug discovery is the drug response exhibited by mice and humans. Hits that prove to be effective in animal models may fail to show effectiveness in human clinical trials. Several factors can impact the translational power of experimental therapies, including the non-reproducibility of the pre-clinical studies, the technical unfeasibility, and the poor prediction efficacy of animal models for drug safety in humans (113, 115). Although several compounds have already been tested using these approaches, only a very small number of them have thus far cleared their way to clinical trials.

An important issue to be addressed during a drug development program is the dose conversion from mice to humans (116). In fact, extrapolation of the dose from the animal model in question to humans is a crucial step that has to take into account different aspects such as varied pharmaco- kinetic and dynamics, in body weight and surface, and in the overall physiology existing between the organisms. To overcome this, different approaches for dose conversion have been described (117). In particular, the US Food and Drug Administration released guidelines for industry based on the dose conversion approach namely “dose by factor” that represents an empirical method based on the use of the No Observed Adverse Effect Levels to derive the maximum recommended starting dose for a clinical trial (118). Other approaches are specifically applied for targeted model systems. For example in the case of mouse-human drug conversion, these are based on the comparison, the similarity, or they can be pharmacokinetics-guided (118).

Based on these aspects, we therefore propose a three-step functional study and pharmacological testing pipeline (**Figure 3**). Step-1 requires identification of a potentially causal gene or gene variant that could act as a putative drug target. This will be used in step-2, where complementary functional studies will be performed by mouse (A), *in vitro* cell culture (B), and zebrafish (C) labs to gather data. These model systems are commonly tested sequentially following the identification of potential *in silico* hits, starting with cells followed by animal models including teleost or bony fish (zebrafish and medaka) and mammals. However, their parallel use will increase output and validation prior to testing in higher animal models and human clinical trials. If the putative target shows compelling data in multiple model systems, step-3 will be performed to discover new compounds that could act on the putative target or its pathway. *In vitro* cultured cells and zebrafish will act as primary complementary testing platforms for therapeutic discovery using compound(s) that could hypothetically act on the candidate protein or tractable candidates within the signaling pathway. These lists of compounds could be derived from *in silico* drug docking of synthetic chemical structures (e.g., by using 3D protein crystal structure), literature-based drug repurposing, or pre-made commercially available chemical libraries predicted to act on the signaling pathway. Both zebrafish (larvae or *ex vivo* cultured scales) and *in vitro* systems allow testing of these ‘low-confidence’ candidates on a (semi-)high throughput scale. When a sub-set of these compounds shows a pre-defined effect (validation) in both systems, these ‘higher confidence’ compounds with an osteogenic potential can then proceed to stage-2 where they can be further validated and tested in the terrestrial mouse system and/or adult zebrafish (**Figure 3**). This pipeline requires cross-discipline collaboration between several laboratories exercising their expertise. This will speed-up novel drug discovery, limit false-positive findings (since multiple labs will validate the findings independently), and reduce unnecessary animal use.

## From Genetic Discoveries to Improved Patient Management in Rare Bone Diseases

The path from discovery of the underlying genetic cause of a disease through to the development of a clinical therapy is a long and winding road, but some success stories have been seen during the recent years in the field of rare bone diseases, such as targeted therapies for HPP (119), X-linked hypophosphatemic rickets (120) and achondroplasia (121).

In the field of bone mass disorders, an example of promising treatment discovery is represented by the OPTA2, as described above, a rare monogenic disease characterized by bone fragility despite increased bone mass (122). The phenotype of OPTA2 has been characterized since the beginning of the 20th century, then in the 2000s, mutations in the *CLCN7* gene were associated with the disease (123). The *in vitro* characterization of the pathogenic variants revealed that *CLCN7* mutations negatively affected osteoclast function, impairing their ability to resorb bone matrix. Although the number of osteoclasts is normal or even increased, they were not able to properly acidify the resorption lacuna and remove mineralized bone matrix (122, 124). The first animal model for OPTA2 carrying the mouse homolog of the most frequent and well characterized human *CLCN7* mutation (p.G215R) was created in 2013 (125). Interestingly, the mouse model not only recapitulated the bone defect but also allowed deep phenotyping of the disease, revealing its complex multiorgan involvement (126). To date, there has been no disease-specific therapy and patients receive only symptomatic management. Interferon gamma (Actimmune), a promising non-targeted therapeutic approach, was tested in a phase 2 clinical trial (NCT02584608). Unfortunately, despite the encouraging results obtained from the animal model (127), the clinical trial in OPTA2 patients was not successful (128). However, availability of reliable *in vitro* and *in vivo* models, and the known dominant negative nature of the disease-causing mutation, allowed the development of another innovative experimental OPTA2 targeted therapy, based on the systemic delivery of mutation-specific siRNA (73). This experimental approach has proven to be effective in pre-clinical studies, rescuing both the skeletal and extra-skeletal defects with no safety issues (73, 126). This therapy’s clinical development is expected in the coming years. Therefore, the OPTA2 story is an example of a “canonical” pipeline in the study of a monogenic condition that started from the discovery of the disease causing-mutation and hopefully will end with the clinical development of targeted therapy.

Unravelling the pathogenic mechanisms underlying rare monogenic conditions can result in novel treatment options not only for the specific rare disease but also for more common disorders. Clear illustrative examples of this are sclerosteosis and van Buchem disease, two related conditions arising from mutations affecting the *SOST* gene which codes the protein sclerostin (24–27, 129, 130). Sclerostin presented as an interesting drug target for osteoporosis, not only based on its function but also because of its expression pattern, being almost exclusive to bone. Furthermore, it became clear that modulation of sclerostin concentration and activity affects bone mass, as indicated by the fact that *SOST* SNVs are associated with BMD (131) and that heterozygous carriers for mutations causing sclerosteosis and van Buchem disease also show an increased bone mass (132). Clinical trials with a humanized monoclonal antibody that targets sclerostin (romosozumab) have shown that the drug increases bone formation and decreases bone resorption in postmenopausal women with low BMD. In 2019, romosozumab was approved as an important anabolic treatment for severe osteoporosis (133). Yet, no long-term data on romosozumab treatment efficacy or safety is available (134). Concerns have indeed been raised regarding the increased risk of cardiovascular events following romosozumab treatment in older women (135). This is not surprising as canonical WNT signaling plays a dominant role also in other pathologies, including heart disease such as myocardial infraction, cardiovascular remodeling, and congestive heart failure (136). Elevated sclerostin levels have been positively associated with increased arterial calcification, a physiological adaptation to inhibit atherosclerosis development (137). The effect of sclerostin on vascular pathophysiology has been reviewed in detail elsewhere (138). However, this brings to light a crucial caveat in romosozumab treatment that requires further attention and consideration.

These two examples illustrate that knowledge on pathogenic mechanisms underlying very rare conditions is crucial for significant advances in patient care in common disease. Only hypotheses based on scientific evidence can move the field forward, as has been demonstrated by the development of targeted therapies for HPP (119), X-linked hypophosphatemic rickets (120) and achondroplasia (121). The recent advances in genetic and molecular studies, and access to animal models are likely to decrease the time interval from disease characterization to development of targeted therapies. Importantly, the knowledge obtained from rare monogenic disorders of low and high bone mass can be used to advance therapeutic discoveries for more common disorders, especially osteoporosis.

High-throughput sequencing technologies have led to a significant increase in knowledge in the area of rare hereditary diseases (139). At the same time, methodological developments have revolutionized biological science by allowing large-scale cost-efficient sequencing, frequently integrated with proteomics, transcriptomic and other omics in a multi-omics approach. On the other hand, such data can be merged with clinical data, phenotyping information, imaging data, family history details, and biochemical results in a multi-source highly integrated data analysis process (140, 141). Combining all the available data, bioinformatics methods and Artificial Intelligence approaches with machine learning, has the potential of creating a paradigm shift in the gene discovery and consequently, in the entire diagnostic pathway (142–144).

**Figure 4:**
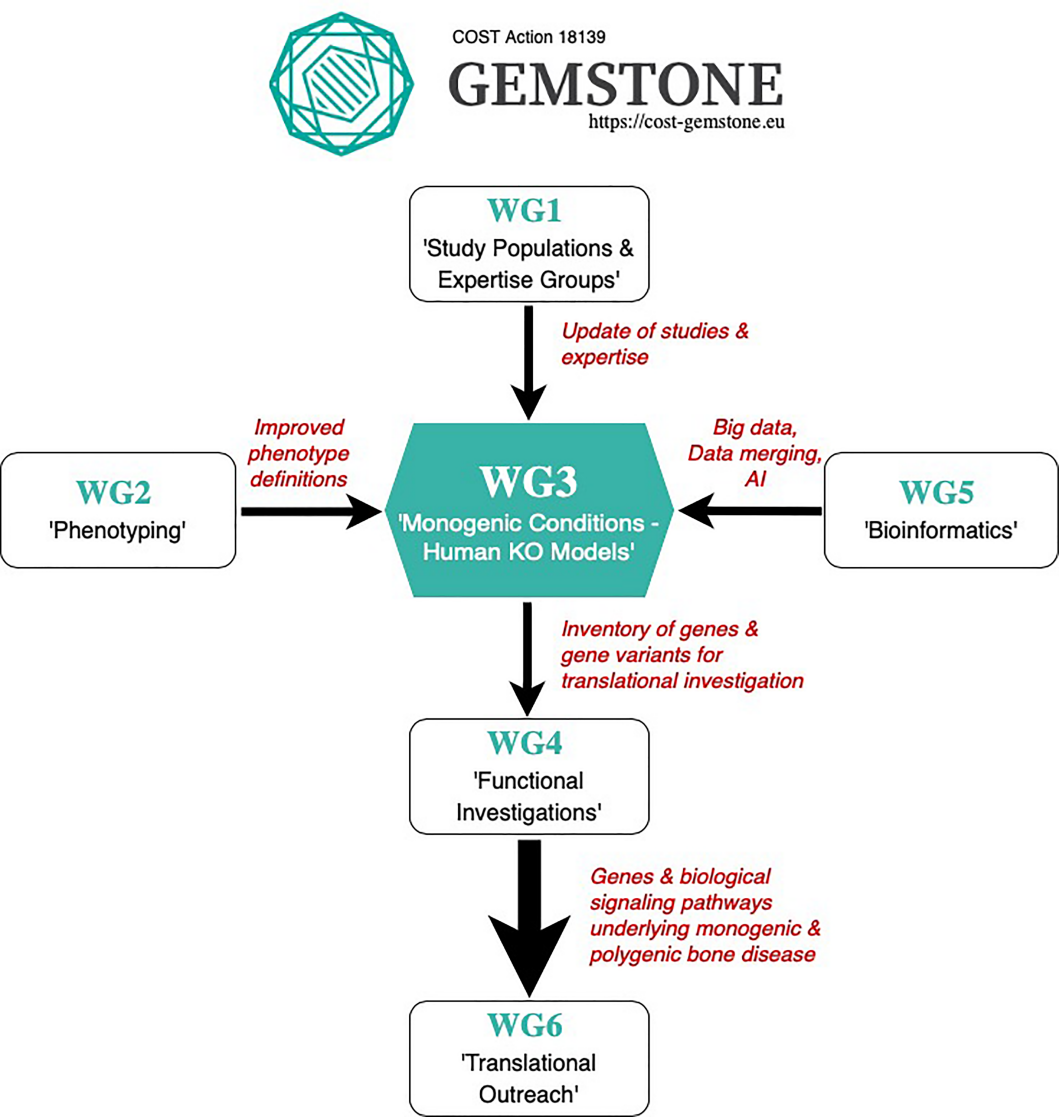
GEMSTONE Working Group 3 Proposed Roadmap.

Studies of rare conditions, more than any other disease, need structures for data integration between academia and clinical institutions in order to collect a sufficient number of patients from which patterns can emerge to underpin valid research questions. Success in such a collaborative multi-center approach relies on proper phenotyping with standardized disease classification, unified and reliable data capture, merging of data, analyses processes, and storage methods (145, 146). In addition to this, the quality of machine learning outputs and bioinformatics methods strictly depends on the quality and consistency of input data. Considering all the mentioned points, a goal of the GEMSTONE Working Group 3 is to create a roadmap for gene discoveries in the context of rare bone diseases, which requires an established process in close collaboration with all other GEMSTONE Working Groups (**Figure 4**) to assure data quality and impact.

## Conclusions

Although individually rare, genetic disorders of the skeleton are of clinical relevance because of their combined overall frequency and impact. A correct diagnosis has important implications in genetic counselling when determining the mode of inheritance and the recurrence risk in affected families. Furthermore, a specific diagnosis is helpful in the follow-up and treatment of the patient, and in predicting long-term outcomes of the disorder. As highlighted in this review, the clinical and genetic spectrum of rare disorders of low and high bone mass are still inadequately understood and novel monogenic disorders still remain to be discovered. Such disorders have the potential of providing novel information about molecular pathways regulating the numerous and only partially known aspects of bone metabolism. New gene discoveries need to be extensively validated and explored in various *in vitro* and *in vivo* experiments. All this work has the potential to lead to significant scientific advancements that can be translated into improved patient care for individuals affected by rare bone diseases, and also more widely for the millions of individuals affected by osteoporosis.

## GEMSTONE Working Group 3 COST Action Members

Susanna Balcells, J. H. Duncan Bassett, Dylan J. M. Bergen, Eveline Boudin, Maria Luisa Brandi, Alper Cebi, Jannie Dahl Hald, Emma Duncan, Melissa M. Formosa (WG3 Leader), Natalia Garcia-Giralt, Celia L. Gregson, Daniel Grinberg, Gretl Hendrickx, Wolfgang Högler, Anders Kämpe, Douglas Kiel, Outi Mäkitie (WG3 Leader), Antonio Maurizi, Georgina McDonald, Maria Carolina Medina Gomez, Evangelia Ntzani, Diana Ovejero Crespo, Jose A. Riancho, Luca Sangiorgi, Uunur Styrkársdóttir, Anna Teti, Wim Van Hul, Graham R. Williams, Angela Xuereb-Anastasi, Wei Zhou, M Carola Zillikens

## Author Contributions

OM and MMF initiated the manuscript. DB, AK, MMF, and SB generated the figures for the manuscript. All authors contributed to the article and approved the submitted version.

## Funding

Funding was obtained from the GEMSTONE COST Action (CA18139). MMF has received research funds from The University of Malta Research, Innovation and Development Trust and The Malta Community Chest Fund, and the Research Excellence Programme (REP-2020-011; Project GeOM financed by the Malta Council for Science & Technology, for and on behalf of the Foundation for Science and Technology, through the Research Excellence Programme). DB is funded by the Foundation Fellowship from Versus Arthritis (Grant no. 22044). NG-G and DC are supported by CIBERFES (CB16/10/00245 [ISCIII]), FEIOMM2019 and European Regional Development Fund (ERDF). WZ is funded by the Jaap Schouten Foundation (The Netherlands). WVH received a Methusalem-OEC grant for the project “GENOMED” and a Research Fund of the University of Antwerp (FFB190208). MB has received funding from La Fondazione Italiana Ricerca sulle Malattie dell’Osso. The Origins of Bone and Cartilage Disease Programme analysed the skeletal phenotype of knockout mice generated by the International Mouse Phenotyping Consortium (IMPC) and was funded by a Wellcome Trust Strategic Award (101123) to GW and JB. OM is funded by the Sigrid Jusélius Foundation. DG and SB declare that this study received funding from MINECO (SAF2016-75948R and PID2019-107188RB-C21). The funder was not involved in the study design, collection, analysis, interpretation of data, the writing of this article or the decision to submit it for publication.

## Conflict of Interest

MZ has received honoraria in the past for lectures or advice from Alexion, Amgen, Eli Lilly, Kyowa Kirin, Shire and UCB, unrelated to the current work. WH has received research support by Alexion, Kyowa Kirin, Ultragenyx and Internis Pharma. AM is co-inventors of the patented siRNA treatment for the ADO2 therapy mentioned in the article. MB has received honoraria from Amgen, Bruno Farmaceutici, Calcilytix, Kyowa Kirin; Academic grants and/or speaker: Abiogen, Alexion, Amgen, Bruno Farmaceutici, Echolight, Eli Lilly, Kyowa Kirin, MSD, NPS, Servier, Shire, SPA, Theramex, and acts as a consultant for Alexion, Bruno Farmaceutici, Kyowa Kirin, Servier, Shire, unrelated to the current work.

The remaining authors declare that the research was conducted in the absence of any commercial or financial relationships that could be construed as a potential conflict of interest.

## Publisher’s Note

All claims expressed in this article are solely those of the authors and do not necessarily represent those of their affiliated organizations, or those of the publisher, the editors and the reviewers. Any product that may be evaluated in this article, or claim that may be made by its manufacturer, is not guaranteed or endorsed by the publisher.
